# Correlation between annual activity patterns of venomous snakes and rural people in the Niger Delta, southern Nigeria

**DOI:** 10.1186/1678-9199-19-2

**Published:** 2013-02-27

**Authors:** Godfrey C Akani, Nwabueze Ebere, Daniel Franco, Edem A Eniang, Fabio Petrozzi, Edoardo Politano, Luca Luiselli

**Affiliations:** 1 Department of Applied and Environmental Biology, Rivers State University of Science and Technology, Port Harcourt, Rivers State, Nigeria; 2 Planland, Studio Tecnico Daniel, Franco, Rome, Italy; 3 Department of Forestry and Wildlife Sciences, University of Uyo, Akwa-Ibom State, Nigeria; 4 Environmental Studies Centre Demetra, Rome and Fano, Italy

**Keywords:** Monthly activity, Venomous snakes, Rural people, Nigeria

## Abstract

**Background:**

Venomous snakes are among the most serious health hazards for rural people in tropical regions of the world. Herein we compare the monthly activity patterns of eight venomous snake species (Elapidae and Viperidae) with those of rural people in the Niger Delta area of southern Nigeria, in order to identify the periods of highest potential risk for persons, and the human group actually at greater risk of snakebite.

**Results:**

We documented that above-ground activity of all venomous snakes peaked in the wet season, and that high snake activity and high human activity were most highly correlated between April and August. In addition, we documented that women and teenagers were at relatively higher risk of encountering a venomous snake than adult males, despite they are less often in the field than men.

**Conclusions:**

Our results suggest that future programs devoted to mitigate the social and health effects of snakebites in the Niger Delta region should involve especially women and teenagers, with ad-hoc education projects if appropriate. We urge that international organizations working on social and health problems in the developing world, such as IRD, DFID, UNDP, should provide advice through specific programs targeted at especially these categories which have been highlighted in comparatively potential higher threat from snakebites than adult men.

## Background

Venomous snakes certainly are among the main health hazards for rural people in tropical regions of the world, while it is calculated that many thousands of people die yearly on account of snakebite, especially in India, but also in Africa and South America [1,2].

Several independent factors may increase the probability of being bitten and eventually killed by a snake in a tropical area. Firstly, the antipredatory behavior of a snake species certainly influences the likelihood of a bite: for instance, the puff adder (*Bitis arietans*) is more inclined to bite than a Gaboon viper (*Bitis gabonica*); thus, despite similar ecologies (but not habitat selection) and venom compositions and potency, the former is more likely to kill people than the latter [2,3]. Secondly, in geographic areas where there is a higher diversity of venomous snakes, the probability of being killed by snakebite is greater than in areas where there are fewer dangerous snakes. So, for instance, the probability of experiencing a fatal bite is certainly higher in India or in several regions of tropical Africa (where many sympatric highly venomous snake species do exist) than in South America. Thirdly, geographic areas with a greater density of people working in rural activities are more prone to be affected by high rates of fatal bites than areas where the rural human density is low. Fourthly, the probability of being bitten in a given tropical area cannot be constant across the year, but should depend on the monthly activity patterns of snakes and on correlating the activity patterns between snakes and humans [2]. Indeed, it has been demonstrated that in tropical areas three quarters of the bites happen during agricultural tasks, hunting or while walking to or from work, and are hence linked to occupational activities [2].

In the present paper, by taking advantage of the most prolonged longitudinal field study (16 years) on the ecology of tropical snake communities available in the world [4,5], we analyze the correlation between the monthly activity patterns of eight species of venomous snakes (belonging to the families Elapidae and Viperidae) and people in rural areas of the Niger Delta, in southern Nigeria. This study region is particularly interesting from the venomous-snake point of view because, having been extensively deforested in the last 50 years, it has been invaded by the spitting cobra (*Naja nigricollis*), which otherwise tends to inhabit the savannah, to the detriment of the once dominant cobra species (the forest cobra *Naja melanoleuca*), typically adapted to life in the rainforest [3,6-11].

As to Nigeria, the only detailed data on the epidemiology and occurrence of venomous snakebites came from the savannah region in the north of the country [12,13]. These studies revealed an incidence (per 100,000 inhabitants) of 48–603 bites (mean = 160), with a morbidity of 100–150, a lethality in hospitals of 2.1-27, and an overall mortality of 15.6 [2]. However, no such data are available for the southern portion of the country, where the main vegetation zone is constituted by mangroves and rainforest [7]. Our study aims to verify, inside the rainforest vegetation zone of Nigeria, the specific period of the year in which the greatest activity of venomous snakes occurs in the field, and which period of the year presents the highest correlation between occupational activities of people and above-ground activity of snakes.

## Methods

### Study area

**Figure 1:**
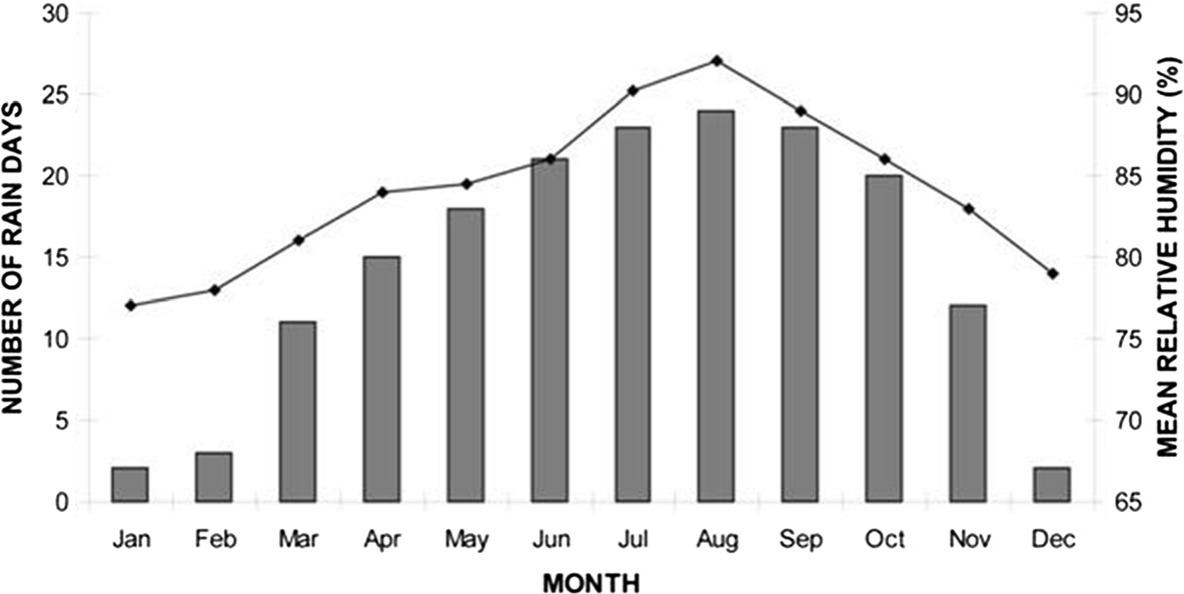
Pluviometric patterns in the study area.

The study was conducted during field surveys between September 1996 and June 2011, in the plantation-bushland-secondary-forest mosaic region of the Niger Delta, in southern Nigeria. We conducted our surveys in eight states of the Federal Republic of Nigeria, from West to East: Delta, Bayelsa, Anambra, Imo, Rivers, Abia, Akwa-Ibom, and Cross-River. Thus, we surveyed an irregular surface of approximately 400 × 250 km. The study area, which is heavily populated with hundreds of villages interspersed by patches of forests and cultivated lands, is especially important for the economy of Nigeria because of its large-scale oil extraction and liquefied natural gas transmission installations [7]. The forest patches may be dry-land or swamp rainforest type. Mangrove forests (*Avicennia* spp., *Rhizophora racemosa*) are the dominant vegetation types in the areas of the fluvial systems influenced by salty or brackish water. The climate of the study region is tropical sub-Saharan, with well delineated dry and wet seasons and relatively small monthly fluctuations between maximum and minimum temperatures [14]. The monthly pluviometry of the study area is given in Figure 1.

### Measurement protocol of snake activity

Annual snake activity patterns were assessed by visual-encounter surveys (VES) and by counting death-on-road (DOR) specimens, assuming that there should be a linear relationship between intensity of above-ground activity in snakes and their probability of being observed during VES surveys and risk of being squashed along a road [1,15-17]. The effort time (number of days in the field) was recorded. When two (or three) independent observers conducted a VES survey on the same day, the number of days recorded for the analyses was two (or three), given that there was total independence of observations and no possibility of erroneously replicating the data.

Survey time was typically from 7h00 to 11h00 and from 16h00 to 19h00, whereas searches were generally suspended during midday because of reduced snake activity at that time. Nocturnal surveys were generally avoided due to safety reasons.

### Rural human activity

To compare the monthly patterns of activity between snakes and rural persons, we constructed an empirical index of the frequency of encountering of people in the field by month, during the snake surveys. The following human activities were considered: walking, farming, fishing, hunting, canoe-carving, logging, and palm-oil production. This index was calculated during the year 2011, and was assumed to be relatively constant over the years.

### Statistical analyses

To evaluate whether each of the species (and the whole of the species at once) was observed at a similar frequency of occurrence throughout the various months, the various records were grouped into monthly intervals (from the first to the last day of each month). Then we determined the relative sampling per monthly interval by dividing the number of days spent in the field in each month by the total number of days in the field during the entire research period (Table 1). Using a null hypothesis of equal distribution frequency among months, we then generated the number of snakes expected in each month by multiplying the total number of snakes found during the study by the relative sampling for each monthly interval. Finally, the observed and expected values were compared by the chi-squared test.

**Table 1:** Summarized data on the monthly number of field days and relative index of sampling effort used to estimate the expected monthly number of snakes

	**Number of days in the field**	**Relative index**
Jan	144	0.0775
Feb	143	0.0770
Mar	151	0.0813
Apr	156	0.0840
May	155	0.0835
Jun	149	0.0802
Jul	162	0.0872
Aug	177	0.0953
Sep	161	0.0867
Oct	142	0.0765
Nov	150	0.0808
Dec	167	0.0899
**Total**	1857	

### Analyses for correlation

Although not an entirely rigorous comparison between the activity patterns of snake and rural people, we constructed a frequency index showing the overlap between the empirical index (mean number of people/hectare), as an annual percentage (%) for men, women and teenagers; and the % index (also on a yearly basis) of the number of snake observations according to species. In this graphical comparison, it should be emphasized that the human index is related to a surface (hectare), contrary to the ophidian index.

## Results

### Activity patterns of venomous snakes

This study focuses on eight venomous ophidian species, four from Elapidae (the spitting cobra *Naja nigricollis*, the forest cobra *Naja melanoleuca*, the tree cobra *Pseudohaje goldii* and Jameson’s green mamba *Dendroaspis jamesoni*) and four from Viperidae (the Gaboon viper *Bitis gabonica*, the nose-horned viper *Bitis nasicornis*, the green bush viper *Atheris squamiger* and the West African night adder *Causus maculatus*). Other venomous snakes found in the Niger Delta include: *Toxicodryas blandingii, Toxicodryas pulverulenta, Thelotornis kirtlandii, Dispholidus typus, Crotaphopeltis hotamboeia* and *Atractaspis* spp. [15,16,18]. However, we decided not to analyze these species either because they are only mildly venomous (e.g., *Toxicodryas* spp. and *Crotaphopeltis hotamboeia*), or they are very rare (*Dispholidus typus*), or they are back-fanged and hence unlikely to pose a real threat to people (*Thelotornis kirtlandii*), or because they are subterranean, hence being difficult to analyze reliably as to their monthly activity patterns (*Atractaspis* spp).

**Figure 2:**
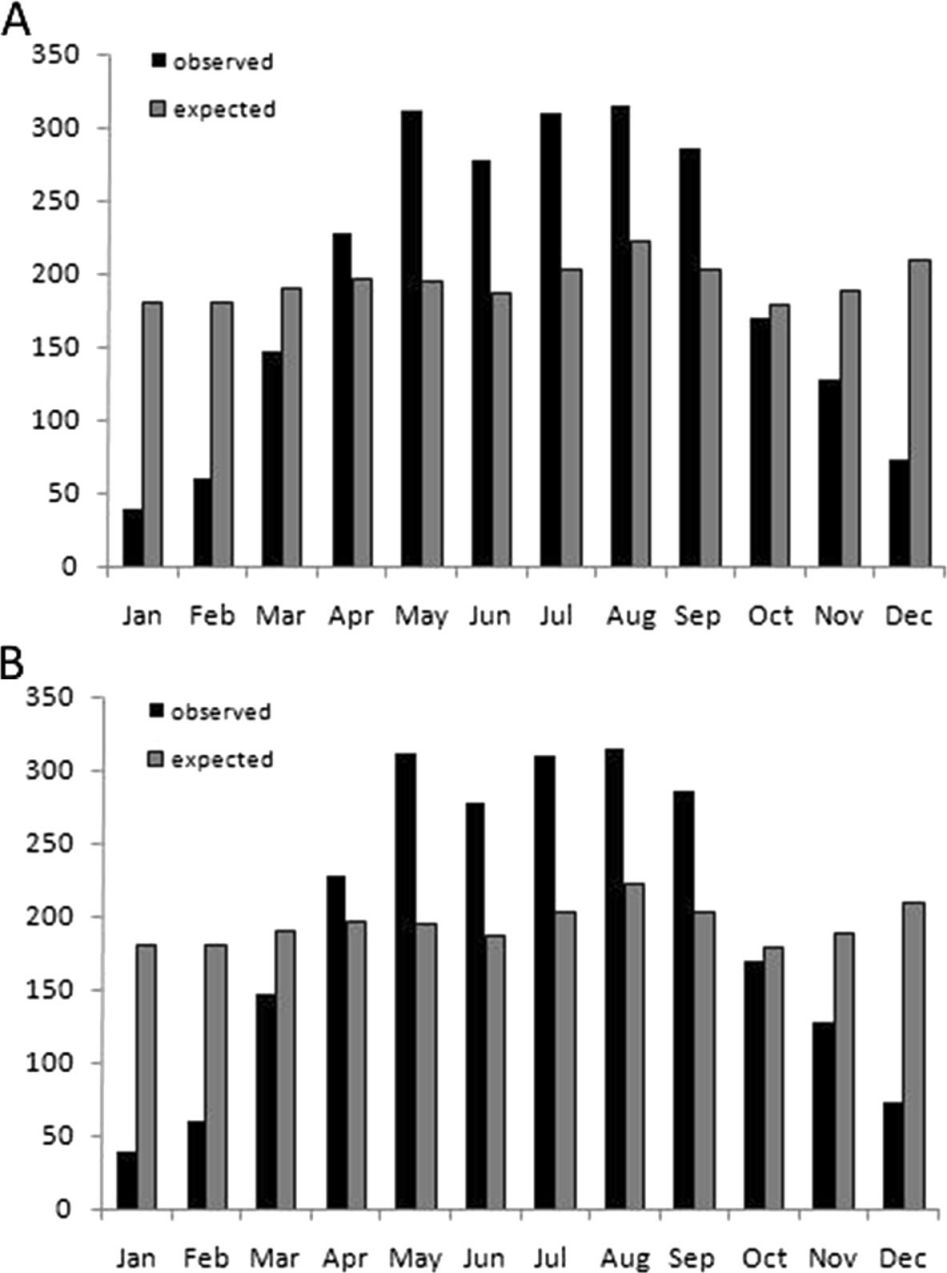
Activity patterns of (A) Elapidae and (B) Viperidae in the study area.

Overall, both the Elapidae (χ^2^ = 557.6, df = 11, p < 0.0001) and the Viperidae (χ^2^ = 902.9, df = 11, p < 0.0001) had significantly uneven activity patterns, with above-ground activity peaking during the wet season (Figure 2). More in detail, the Elapidae showed significantly higher than expected activity from May to September, and significantly less than expected activity between November and February (Figure 2A). The activity presented by Viperidae was significantly greater than expected from April to August, but significantly lower than what was expected between November and March (Figure 2B).

**Figure 3:**
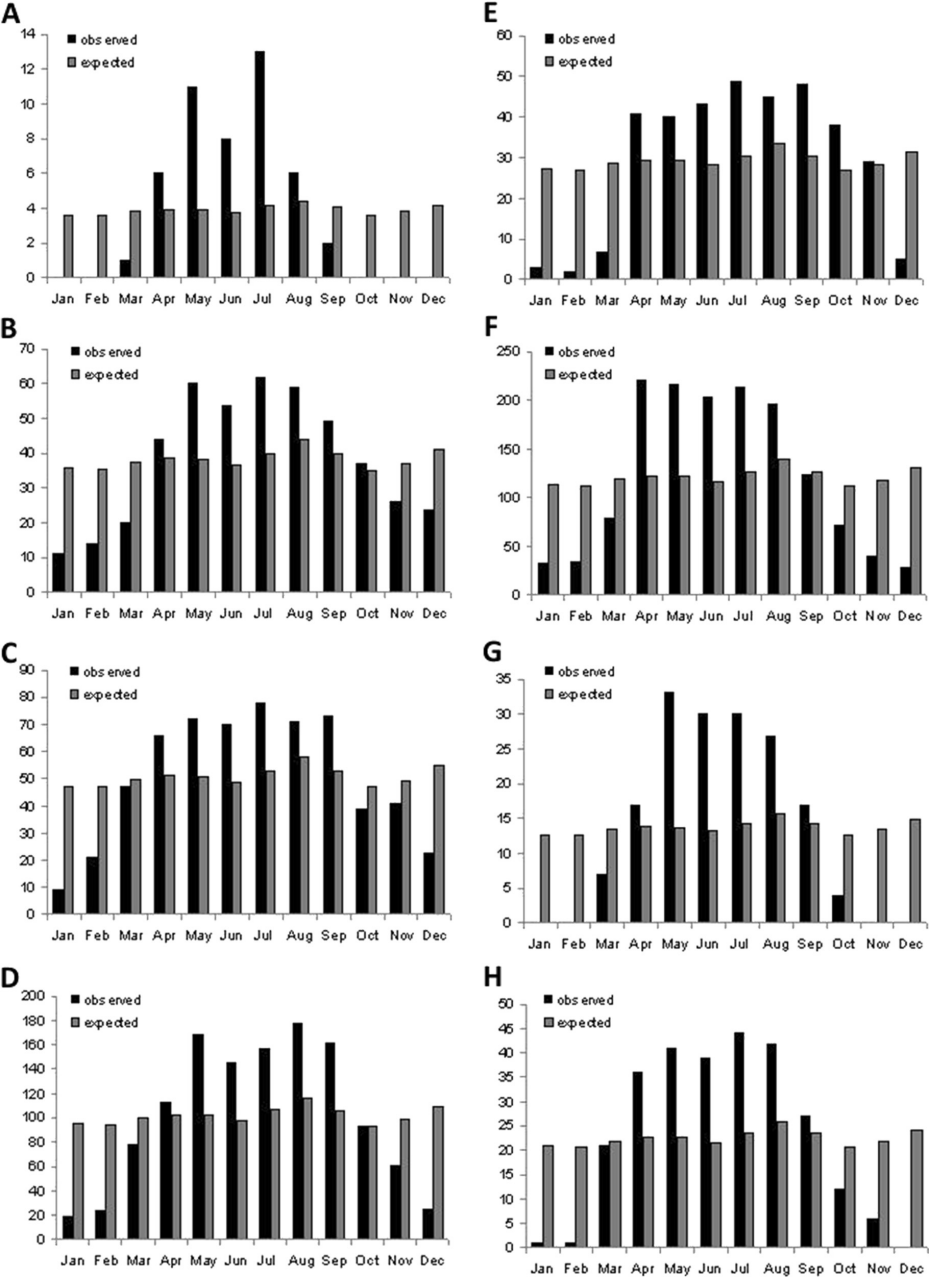
**Activity patterns of the eight snake species in the study area: (A) *Pseudohaje goldii*, (B) *Dendroaspis jamesoni*, (C) *Naja melanoleuca*, (D) *Naja nigricollis*, (E) *Atheris squamiger*; (F) *Causus maculatus*, (G) *Bitis nasicornis*, and (H) *Bitis gabonica.***

The observed-versus-expected monthly numbers of individuals of the eight species of venomous snakes studied herein are given in Figure 3. All species of both families showed a consistent pattern, with significantly more activity than expected during the wet months and significantly depressed activity during the dry months (Table 2). The interspecific differences were very minor, and thus do not need to be discussed here.

**Table 2:** Statistical details of the comparison between observed and expected numbers of snakes

**Species**	**Χ**^**2**^	**Df**	**p value**
*Atheris squamiger*	129.7	11	< 0.00001
*Bitis gabonica*	142.6	11	< 0.00001
*Bitis nasicornis*	136.9	11	< 0.00001
*Causus maculatus*	574.1	11	< 0.00001
*Dendroaspis jamesoni*	88.9	11	< 0.00001
*Naja melanoleuca*	110.7	11	< 0.00001
*Naja nigricollis*	349.6	11	< 0.00001
*Pseudohaje goldii*	60.4	11	< 0.00001

### Activity patterns of rural persons

The human activity with the clearest seasonality pattern was farming, which represents the economic base of the Niger Delta region, and traditionally includes land clearing, preparation, planting, weeding, tending and harvesting as well as post-harvest handling, processing and storage [19]. The farming areas fall into the lowland rainforest zone, where there are relatively significant amounts of dry land. Land clearing is carried out during the dry season usually between December and April and requires 24–40 man days per hectare. During the clearing process, burrowing and crawling animals like vipers, pythons, cobras, tortoises, and rodents are exposed and killed (and a total of 60-100/hectare of such animals may be observed in the region under study). Obviously, the farmers also face an associated risk of being bitten by venomous snakes. The most frequently encountered species during farming are night adders, spitting cobras and green mambas.

After clearing, there is a four- to six-week drying period followed by burning, which is performed not only to reduce the problem of carting away the felled wood but also to facilitate nutrient recycling [19]. The ground is then prepared according to the crop to be planted. The land clearing and preparation are carried out by the family unit and/or hired labor, whereas planting, weeding and tending are mostly done by women. Harvesting methods are manual and at subsistence level because of inadequate storage facilities: the labor is considerably reduced, and is predominantly done by women and teenagers.

Artisanal or subsistence fishing is another major economic base of the Niger Delta and is important in the coastal areas [19-21]. Fishing is carried out all the year round; venomous snakes (mainly nose-horned vipers, forest cobras and tree cobras) are widely encountered while fishing in the rainforest zone, and are known to occasionally bite fishermen.

Hunting is another viable occupation in the rainforest zone, and is carried out by men and youth (often in small groups) typically in a 3:2 ratio, representing 10-15% of each community. Hunting is performed during the day, at night and overnight. Trapping is also practiced using wire snares with a drift fence, subterranean trap or snap trap, while each trapper maintains an average of 1.5 km of traps with 50–70 wire snares which he inspects daily or at intervals of two or three days. Venomous snakes usually encountered during hunting are Gaboon and nose-horned vipers, green mambas and forest cobras.

Logging is an exclusively male occupation [21]. Because of lack of roads, the commonest and cheapest method of transporting the logs is by the waterways formed by digging canals. Transportation of logs is often associated with the risk of encountering/transporting cobras, mambas and vipers.

Palm oil production is another of the main business activities of the study area. The palm fruit ripens between the late rainy season and dry season. Harvesting is carried out exclusively by men and youth, who climb up to the palm fruit (8–20 meters high) to cut down three to five bunches. Occasionally they encounter venomous snakes (*Dendroaspis jamesoni, Atheris squamiger, Naja nigricollis, Pseudohaje goldii, Thelothornis kirktlandi*, and *Dispholidus typus*), which may be very abundant in local palm oil plantations [22]. In this case, the potential danger due to snakes is not merely because of their bites. Indeed, during our studies we recorded several cases in which the palm oil tappers fell on the ground and died, as they defended themselves against (or simply were terrified by) snakes (especially green mambas) which, just to secure their escape, tried to bite them on the tree.

For fishing, hunting, canoe carving, logging and palm-oil harvesting, it was not possible to obtain any other quali-quantitative outline of the human beings presence in the same areas surveyed for snake frequency. Thus, we quantified the number of active farmers (per ha) by month in the study areas, and assumed a linear relationship between the quantities of farmers and those involved in the other abovementioned activities.

**Figure 4:**
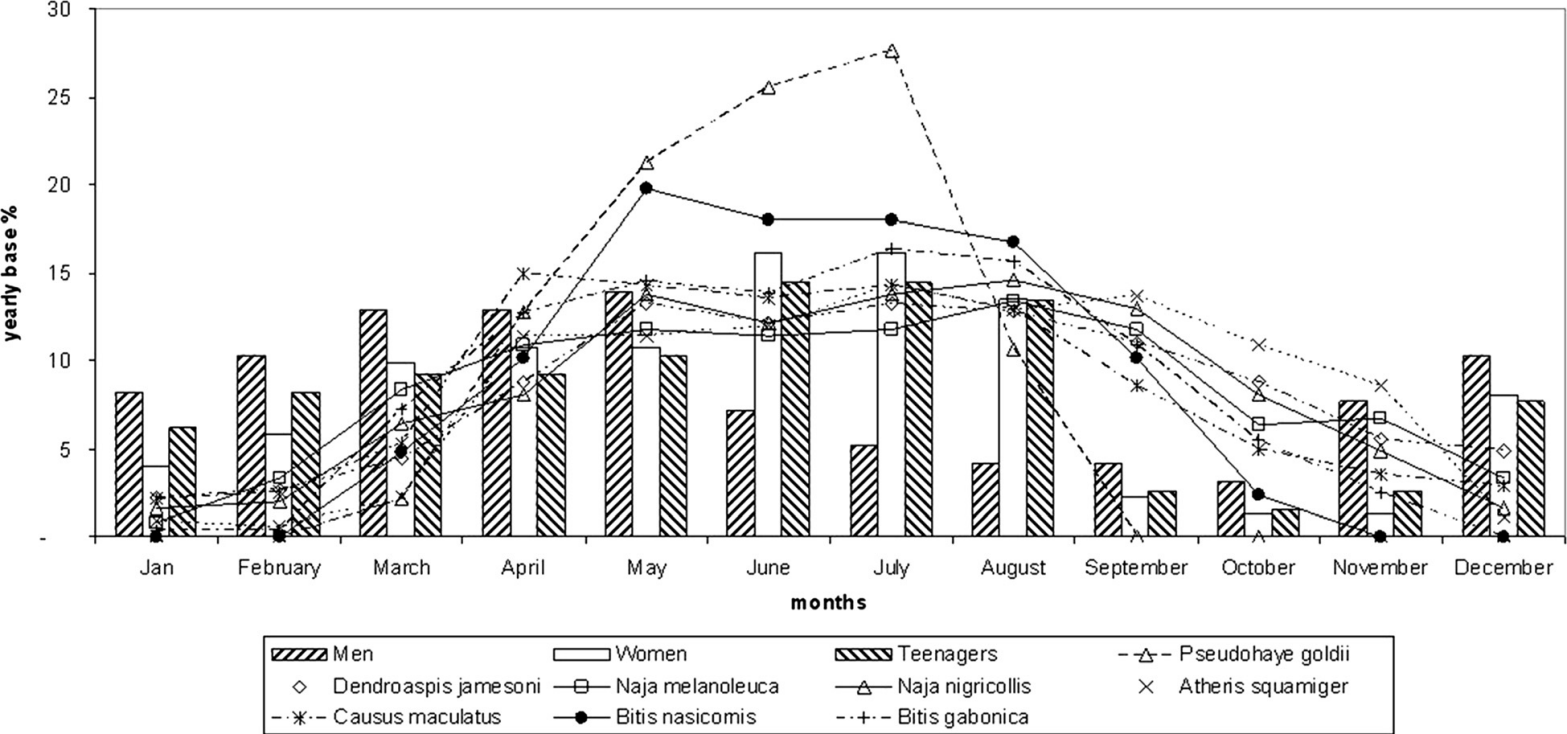
**Matching the activity patterns of rural communities and the eight snake species in the study area.** All data are reported on a yearly basis as a % in each category.

The general trend in the empirical index of humans in the bush throughout the year for this economic activity is outlined in Figure 4. Activities of both women and teenagers in the bush clearly peaked during the wet season months, whereas the activity of men was more even throughout the course of the year.

A comparison of the relative frequency of occurrence of venomous snakes in distinct habitats of the Niger Delta area (Table 3) suggests that, in any case, there should be remarkable variations of the community composition of snake assemblages also at the local level.

**Table 3:** Relative percentage frequency of occurrence of the venomous snake species studied in this paper in three distinct habitats of the Niger Delta, southern Nigeria

**Species**	**Mangroves (n = 160)**	**Palm-oil plantation (n = 284)**	**Swamp forest (n = 129)**
*Dendroaspis jamesoni*	0.63%	9.51	5.4
*Pseudohaje goldii*	6.30%	3.17	
*Naja melanoleuca*	1.26%	7.04	1.6
*Naja nigricollis*	8.13%	8.1	6.2
*Atheris squamiger*		7.04	3.1
*Bitis gabonica*			2.3
*Bitis nasicornis*			7.0
**%**	**16.31**	**34.86**	**25.6**

References: mangroves [25], palm-oil plantation [22], swamp forest [15].

## Discussion

First of all, it is necessary to emphasize that our data are cumulative from several places in the plantation-bushland-secondary forest mosaic, whereas it is well known that the specific composition of snake assemblages may change considerably at the local level, probably due to local environmental conditions and adaptability of each species [15,16,22-25]. For instance, there were remarkable differences in the relative frequency of occurrence of the venomous snakes studied in this paper at three distinct sites with different habitat features of the Niger Delta, with a higher frequency of appearance of target venomous species in palm-oil plantations (34.9% of the total snake specimens encountered) than in mangroves (16.3%) and swamp forest (25.6%).

Interestingly, our study showed that, in the Niger Delta, the highest degree of correlation between the monthly activity patterns of the eight most abundant dangerous venomous snakes and that of rural people was in April-August (wet season), and that women and teenagers were at relatively greater risk than men of encountering a venomous snake during their usual daily practices, although, as expected, the absolute numbers of males at work was higher than that of females or teenagers.

Our first finding was clearly consistent with previous studies: indeed, the seasonal incidence of bites to humans was higher in the rainy season in the Asian agricultural landscapes and in the African savannah, with this pattern being observed in Bangladesh, Mali, Burkina Faso, Nigeria, Ivory Coast, Cameroon, Ghana, and Benin, and even presented a direct relationship with monthly rainfall in Benin, Ivory Coast and Nigeria [2,26]. The second finding was more unexpected, and may reveal that, in the plantation-rainforest mosaic, the group of people more at risk of a snake bite is not, as assumed, adult males [2]. For instance, in the savannah region of northern Nigeria, Warrell et al. [27] demonstrated that young males were the main target for snakebites, especially during the rainy season. However, in that case, most bites were attributable to *Echis ocellatus*, a Viperidae species which does not occur in the rainforest region of southern Nigeria [15,16,18].

Our finding of an expected higher risk of bites for women and teenagers seems to be clinically compelling, as it would be interesting to analyze the hospital records to verify whether our predicted pattern really occurs. Unfortunately, the authors are still in the process of collecting the epidemiological dataset, and thus these noteworthy results could not be corroborated by clinical records. Indeed, according to our present results, it is highly possible that some gender-specific or age-specific human behavior may also reduce the risk of being bitten in the theoretically more exposed groups (women and teenagers). For instance, if women exercise greater cautiousness when entering large plantations than men or teenagers, it would greatly reduce their risk of being bitten. However, given that the body weight of a snakebite victim tends to be positively correlated with the survival rate, it is likely that the mortality incidence should be proportionally higher in women and in teenagers than in adult males [2]. This likelihood that calls for an additional more detailed clinical study to assess the incidence, and the impact on the society, of snakebites in the rural environments of the Niger Delta territory.

## Ethics committee approval

Ethics committee approval was not required according to the Federal Republic of Nigeria laws and our institution rules given the type of protocol used to perform this study.

## Competing interests

The authors declare no conflicts of interests.

## Authors’ contributions

LL provided the idea; GCA, NE, EAE, EP, DF and LL collected and assembled the data; LL and DF analyzed the data; LL and FP drafted the manuscript, and all authors read and approved the final manuscript.
